# Tanshinone IIA reverses oxaliplatin resistance in colorectal cancer through microRNA-30b-5p/AVEN axis

**DOI:** 10.1515/med-2022-0512

**Published:** 2022-07-12

**Authors:** Tingrui Ge, Yonggang Zhang

**Affiliations:** Department of Colorectal Surgery, The First People’s Hospital of Lianyungang, Lianyungang, Jiangsu 222002, P.R. China

**Keywords:** colorectal cancer, Tanshinone IIA, miR-30b-5p, oxaliplatin-resistance

## Abstract

This research aims to explore the role of Tanshinone IIA (Tan IIA) and microRNA (miR)-30b-5p in chemoresistance of colorectal cancer (CRC). The expression levels of miR-30b-5p and apoptosis and caspase activation inhibitor (AVEN) was detected by reverse transcription-quantitative polymerase chain reaction assay. The cell proliferation and apoptosis were examined by 3-(4,5-dimethylthiazol-2-yl)-2,5-diphenyltetrazolium bromide (MTT) and flow cytometry assays. The target relationship between miR-30b-5p and AVEN was confirmed by Dual-luciferase reporter assay. Transwell assay was performed to assess CRC cells’ metastasis. Western blot was carried out to measure the apoptosis-related protein. The results showed that miR-30b-5p was lowly expressed in oxaliplatin-resistance CRC cells SW480 (SW480/R) compared to SW480 cells. Overexpression of miR-30b-5p significantly suppressed the malignant biological behaviors of SW480/R cells and significantly promoted the sensitivity of SW480/R cells to oxaliplatin by down-regulated AVEN expression. Besides, Tan IIA treatment upregulated miR-30b-5p expression in SW480/R cells. Moreover, miR-30b-5p upregulation strengthened the promoting effect of Tan IIA on the sensitivity of SW480/R cells to oxaliplatin. In conclusion, Tan IIA and miR-30b-5p could reverse oxaliplatin resistance of CRC cells and may thus be potential treatment strategies for treating patients with CRC.

## 1 Introduction

Colorectal cancer (CRC) is a common malignant tumor of the digestive system with high morbidity and mortality [1]. CRC is the third most common cancer of all types and the fourth leading cause of tumor-related death [2]. Early clinical manifestation of CRC is not evident before cancer metastasis, which also brings difficulties in the early diagnosis of CRC [3]. The treatment strategies for CRC include surgery, radiotherapy, chemotherapy, molecular targeted therapy, and immunotherapy [4,5,6]. Oxaliplatin, belonging to the family of platinum compounds, is widely used in the treatment of cisplatin-resistant cancers, such as colon cancer and ovarian cancer [7,8]. Although oxaliplatin chemotherapy is commonly used to treat the CRC in hospitals, the emergence of chemoresistance becomes the major clinical hurdle for the CRC treatment.

MicroRNAs (miRNAs) are composed of 19–25 nucleotides and are involved in the regulation of gene expression at the post-transcriptional process by binding to the 3′-untranslated region (3′-UTR) of mRNA of the target gene [9]. Moreover, many research works have revealed that aberrant expression of miRNAs are involved in modulating various cellular processes related to drug resistance in multiple cancers, for example, miR-205 strengthened chemosensitivity of breast cancer cells to doxorubicin chemotherapy by inhibiting the expression of vascular endothelial growth factor A and fibroblast growth factor 2 [10] and miR-765 promoted the multidrug resistance in gastric cancer cells through targeting basic leucine zipper ATF-like transcription factor 2 [11]. Of note, increasing evidences proved that miR-30b-5p is a tumor suppressor in a variety of tumors. miR-30b-5p repressed the esophageal squamous cell carcinoma [12]. In addition, in lung cancer, miR-30b-5p overexpression could inhibit the progression of lung cancer and enhances cisplatin sensitivity through targeting low-density lipoprotein receptor-related protein 8 [13] and miR-30b-5p functions as a metastasis suppressor in CRC by targeting Rap1b [14]. Nevertheless, the role and mechanism of miR-30b-5p in CRC cells and chemoresistance have not been clarified.

Tanshinone IIA (Tan IIA), a compound isolated from the dried root and rootstock of Salvia miltiorrhiza (Danshen) [15]. Recently, many studies have reported that Tan IIA involved anti-inflammatory, antioxidant, and anticancer activities. Tan IIA was found to induce ferroptosis in gastric cancer cells through p53-mediated solute carrier family 7 member 11 (SLC7A11) downregulation [16]. Tan IIA inhibited the ovarian cancer growth through inhibiting malignant properties and angiogenesis [17]. Tan IIA also reduced CRC cell viability via the promotion of mitochondrial fission by activating c-Jun N-terminal kinase-mitochondrial fission factor signaling pathways [18]. Tan IIA is also known to be involved in chemoresistance in cancer [19,20]. In recent years, a large number of studies have shown that drugs can play a role in tumors by regulating the expression of miRNAs. For example, a recent study revealed that Tan IIA could inhibit breast cancer via regulating miR-125b [21]. However, whether Tan IIA participates in the oxaliplatin resistance in CRC remains unknown. And we envisaged that Tan IIA may inhibit chemoresistance through regulating miR-30b-5p.

Apoptosis and caspase activation inhibitor (AVEN) is essential for its anti-apoptotic function [22,23]. In addition to its anti-apoptotic function, AVEN also acts as a signaling sensor in a pathway activated by mutations in ataxia-telangiectasia during DNA damage [24]. Multiple studies have shown that AVEN plays a cancer-promoting role in tumors [25,26,27,28]. AVEN can also control the sensitivity of cancer cells to chemotherapeutic agents [27,28]. Zhang and Jia indicated that AVEN is a target of miR-30b-5p [29]. However, whether miR-30b-5p could affect CRC through AVEN remains to be studied.

Therefore, this study aims at figuring out whether Tan IIA regulates chemoresistance of CRC via the miR-30b-5p/AVEN axis.

## 2 Materials and methods

### 2.1 Cell culture and treatment

Human CRC cell line SW480 were purchased from the Chinese Academy of Science, Shanghai Institute of Biochemistry and Cell Biology. SW480 cells were maintained in Dulbecco’s modified eagle medium, high glucose (Thermo Fisher Scientific, USA) supplemented with 1% penicillin and streptomycin (Thermo Fisher Scientific, USA) and 10% fetal bovine serum (FBS; Gibco, NY) in a humidified atmosphere of 5% CO_2_ at 37°C. Oxaliplatin-resistant CRC cell lines (SW480/R) were induced by oxaliplatin (Meilun, China). Generally, the oxaliplatin-resistant cell lines were developed from SW480 cells by stepwise exposure to increasing concentrations of oxaliplatin from 0.2 to 2 µM, and the drug-resistant cell lines SW480/R was established after 6 months.

### 2.2 Cell transfection

For regulation of expression of miR-30b-5p, miR-30b-5p inhibitor and negative control oligonucleotide NC inhibitor (miR-30b-5p inhibitor: 5′-AGAACAGUGAAAUUUCCAGUCC-3′ and inhibitor control: 5′-CAGUACUUUUGUGUAGUACAA-3′), mimic of miR-30b-5p (5′-UGUAAACAUCCUACACUCAGCU-3′) and mimic control (5′-UACUGAGAGACAUAAGUUGGUC-3′) were purchased from Ribobio (Guangzhou, China). For overexpression of AVEN, AVEN-plasmid were also purchased from Ribobio (Santa Cruz Biotechnology). All these vectors and reagents were transfected into cells and were grown to 70–80% confluence with Lipofectamine 6000 (Beyotime, China). After incubating for 48 h at 37°C and 5% CO_2_, transfected cells were harvested for subsequent use.

### 2.3 Reverse transcription-quantitative polymerase chain reaction (RT-qPCR)

According to the manufacturer’s protocol, total RNA was extracted from the AR42J cells, respectively, by using TRIzol^®^ (Invitrogen), and RNA was reverse-transcribed into cDNA with the QuantiTect Reverse Transcription Kit (QIAGEN, Valencia, USA). The expression of miRNA was detected by Hairpin-it^TM^ miRNAs Quantitation Kit (GenePharma, Shanghai, China) and the RT-qPCR was carried out using SYBR green reagents (Vazyme, Nanjing, China). The 2^−ΔΔCt^ method was employed to calculate the relative expression levels. The following primers were used in PCR assay: U6: 5′-CGCTTCGGCAGCACATATACTA-3′ (F) and 5′-CGCTTCACGAATTTGCGTGTCA-3′ (R); β-actin: 5′-CCTCGCCTTTGCCGATCC-3′ (F) and 5′-GGATCTTCATGAGGTAGTCAGTC-3′ (R); AVEN: 5′-GCGCCGGTTGAAGATGACA-3′ (F) and 5′-TGCAGAGCTAAGGAGGACACT-3′ (R); miR-30b-5p: 5′-CGCGTGTAAACATCCTACA-3′ (F) and 5′-CAGTGCGTGTCGTGGAGT-3′ (R).

### 2.4 MTT assay

The capability of cell proliferation was tested by MTT assay. After Tan IIA stimulation and transfection, SW480/R cells were made into single-cell suspension and seeded to 96-well plates with 5 × 10^3^ cells per well and incubation with MTT solution (10 μL) for 2 h at 37°C and 5% CO_2_ in the dark. Optical density values were detected at 570 nm using ultraviolet spectrophotometer (Thermo Fisher Scientific, USA).

### 2.5 Cell apoptosis assay

Following trypsinization and centrifugation, 1 × 10^6^ Tan IIA-treated and transfected SW480/R cells were collected and treated with 500 μL of buffering agent containing 5 μL of Annexin V-fluorescein isothiocyanate and 5 μL of propidium iodide (Beyotime, Shanghai, China) at room temperature in the dark for 20 min. Then, cell apoptosis rate was analyzed by flow cytometry (FCM) (Beckman Coulter, Inc., Brea, USA).

### 2.6 Dual-luciferase reporter assay

The complementary sequences between AVEN and miR-30b-5p were predicted using the TargetScan (http://www.targetscan.org/vert_72/). The 3′-UTR of AVEN, which contains miR-30b-5p binding site or mutated target site, was synthesized and cloned into the pGL3-basic plasmid (Promega, Madison, USA) to construct the reporter vector AVEN-WT or AVEN-MUT, and SW480/R cells were co-transfected with the aforementioned reporter vectors and miR-30b-5p mimic or mimic control. After 48 h transfection, dual-luciferase gene reporter system (Promega, USA) was applied for detecting of luciferase activity, and the data were normalized to *Renilla* luciferase activity.

### 2.7 Western blotting

Western blotting was performed by a standard protocol. Total protein from SW480/R cells was extracted using radioimmunoprecipitation assay lysis containing protease inhibitor and then measured with a BCA protein assay kit (Beyotime, Shanghai, China). Equivalent amounts of protein were separated by sodium dodecyl sulfate–polyacrylamide gel electrophoresis and transferred onto polyvinylidene fluoride membranes (Millipore, Billerica, USA). After blocking with 5% non-fat milk solution, these bands were incubated first with antibodies at 4°C overnight, membranes were washed with PBST followed by incubation with the appropriate secondary antibody for 4 h at 4°C. Protein bands were imaged with ECL (Millipore, USA). The following primary antibodies were used: anti-cleaved-caspase3 (cat. no. ab32042; 1:1,000; Abcam), anti-AVEN (cat no. 25846-1-AP; 1:1,000; Proteintech), and anti-GAPDH (cat. no. ab9485; 1:1,000; Abcam). GAPDH was taken as an inherent reference.

### 2.8 Transwell assay

Transwell assay was described previously [30]. Briefly, for invasion assay, cells were seeded in 50 μL of Matrigel-coated upper chamber with a pore (BD, United States), the migration of cells were evaluated in the Transwell chamber without Matrigel coating. Medium without 10% FBS was supplied to the upper chamber, and medium with 10% FBS was supplied to the lower chamber. After 24 h incubation, the migration and invasion cells on the membrane surface were fixed by 4% paraformaldehyde and then stained with crystal violet staining solution. Randomly, five fields were counted by using microscope (Olympus, Japan).

### 2.9 Statistical analysis

All data were analyzed using SPSS 17.0 software. Each experiment was repeated at least three times, and the data were presented as mean value ± standard deviation (SD). Student′s *t* tests or one-way ANOVA analysis followed by Tukey’s test were used for the statistical analysis. *P* < 0.05 was perceived statistically significant.

## 3 Results

### 3.1 The miR-30b-5p expression is downregulated in oxaliplatin-resistant CRC cell

**Figure 1:**
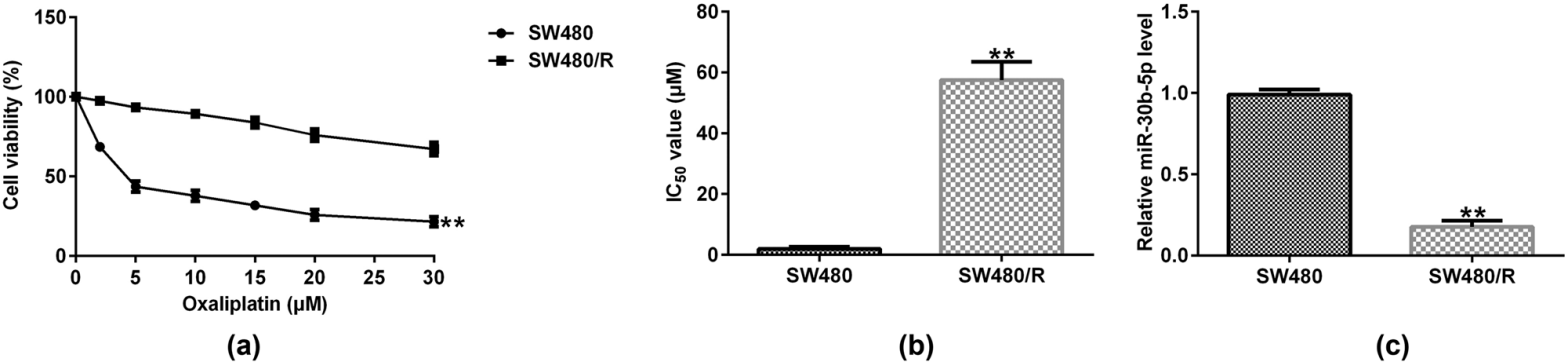
miR-30b-5p expression is downregulated in SW480/R cells. (a) MTT assay was performed to assess cell viability. (b) IC_50_ value of different cells. (c) RT-qPCR analysis of miR-30b-5p expression level. Data are represented as the mean value ± SD of three independent experiments. **P < 0.01 vs SW480 cells.

To explore the potential regulatory role of miR-30b-5p in oxaliplatin-resistant CRC, we established the oxaliplatin-resistant SW480 (SW480/R), and analyzed the sensitivity of oxaliplatin in SW480 cells. MTT assay results showed that cell viability of SW480/R was higher than that of SW480 with the treatment of different concentrations of oxaliplatin from 0 to 30 μM (Figure 1a). The IC_50_ value of SW480/R was higher compared to that of SW480 (Figure 1b). RT-qPCR results unveiled that expression level of miR-30b-5p in SW480/R was lower than that in SW480 cells (Figure 1c).

### 3.2 AVEN was a direct target of miR-30b-5p

**Figure 2:**
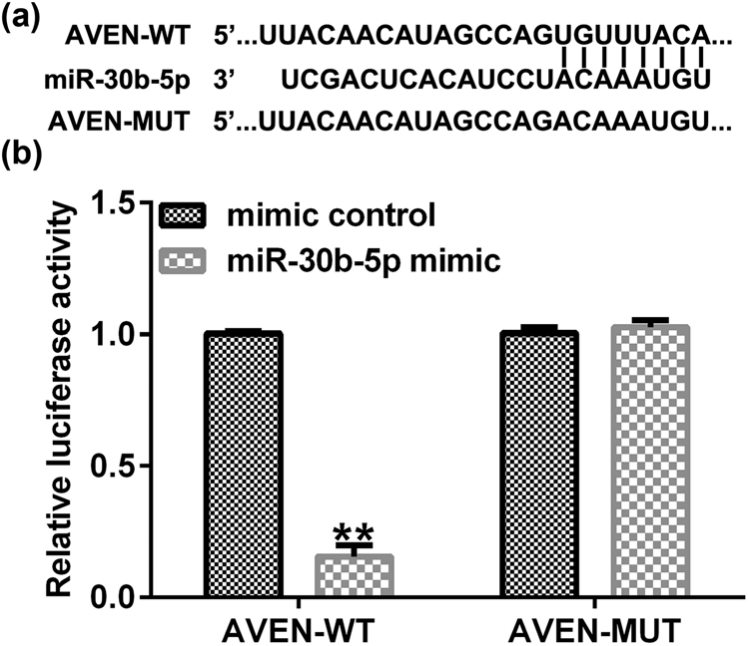
miR-30b-5p directly targets AVEN. (a) The potential binding site between miR-30b-5p and AVEN was predicted by TargetScan website. (b) The dual-luciferase gene reporter assay was completed to confirm the direct binding relationship between miR-30b-5p and AVEN. Data are represented as the mean value ± SD of three independent experiments. **P < 0.01 vs Mimic control group.

Bioinformatics assay was executed to expound the molecular mechanism of miR-30b-5p in oxaliplatin-resistant CRC cell. By using the TargetScan, we discovered that miR-30b-5p harbored the potential AVEN bind sites (Figure 2a). To further determine the relationship between miR-30b-5p and AVEN, dual-luciferase gene reporter assay was carried out in SW480/R cells, the luciferase activity of AVEN 3ʹ-UTR was significantly reduced, when the cells were transfected with miR-30b-5p mimic. However, when the putative binding sites were mutated, the miR-30b-5p mimic exhibited modest effects (Figure 2b).

### 3.3 miR-30b-5p negatively regulates the AVEN in SW480/R

**Figure 3:**
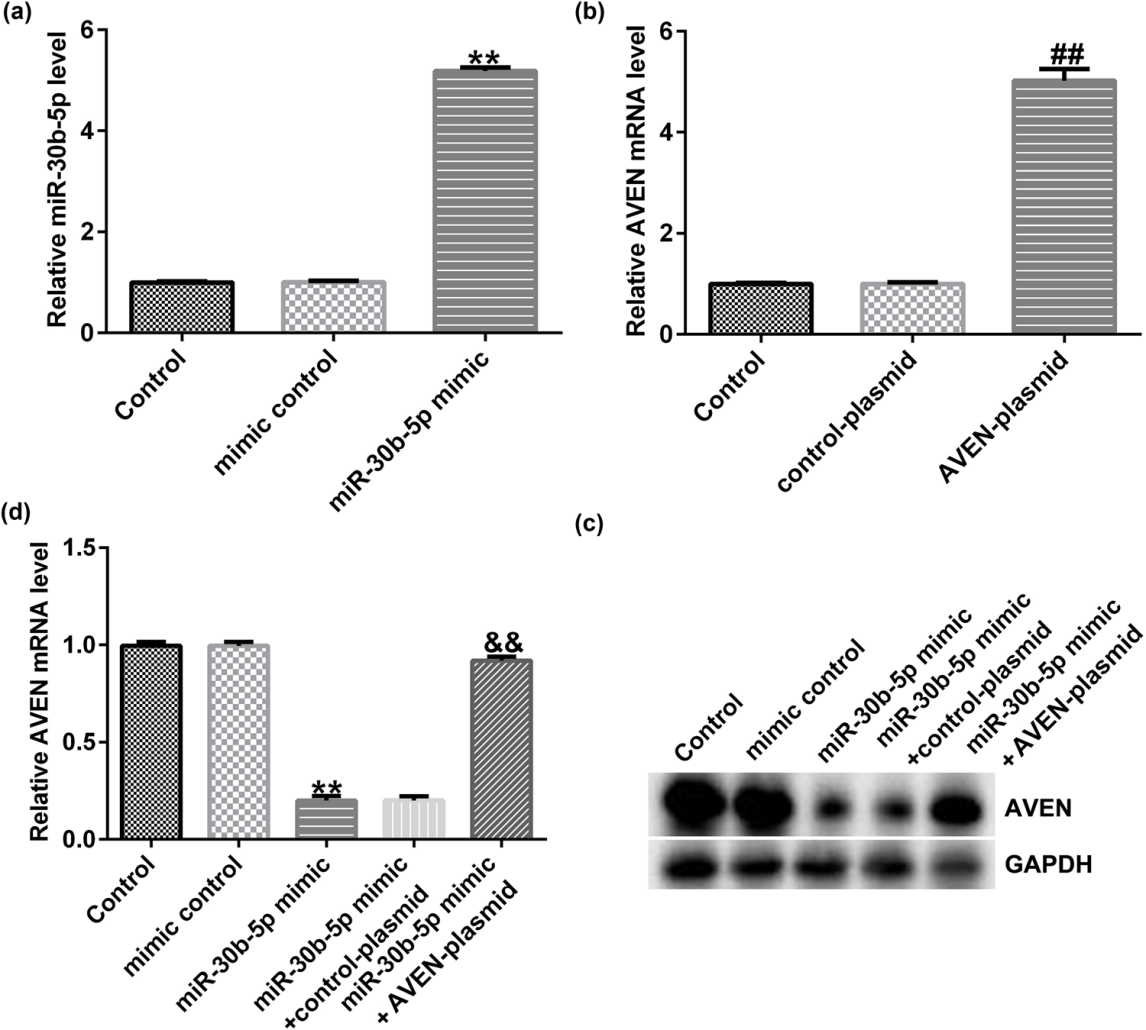
miR-30b-5p negatively regulates AVEN expression in SW480/R cells. (a) The efficiency of miR-30b-5p overexpression was verified by RT-qPCR assay. (b) RT-qPCR was performed to detect the level of AVEN with AVEN plasmid transfection. (c and d) RT-qPCR and Western blot was performed to assess AVEN levels in SW480/R cells following miR-30b-5p overexpression. Data are represented as the mean value ± SD of three independent experiments. **P < 0.01 vs Mimic control; ^##^ *P* < 0.01 vs control-plasmid group; ^&&^ *P* < 0.01 vs miR-30b-5p mimic + control-plasmid.

RT-qPCR analysis certified that miR-30b-5p expression level in SW480/R cells was upregulated upon miR-30b-5p mimic transfection, compared with that in the mimic control group (Figure 3a). RT-qPCR result showed that AVEN-plasmid transfected into SW480/R could markedly upregulated its expression level in contrast with control-plasmid groups (Figure 3b). Furthermore, we clarified that overexpression of miR-30b-5p had markedly decreased the mRNA and protein levels of AVEN in SW480/R cells, while co-transfection of AVEN-plasmid significantly reversed these phenomenon (Figure 3c and d).

### 3.4 MiR-30b-5p downregulated AVEN to inhibit SW480/R proliferation and oxaliplatin resistance

**Figure 4:**
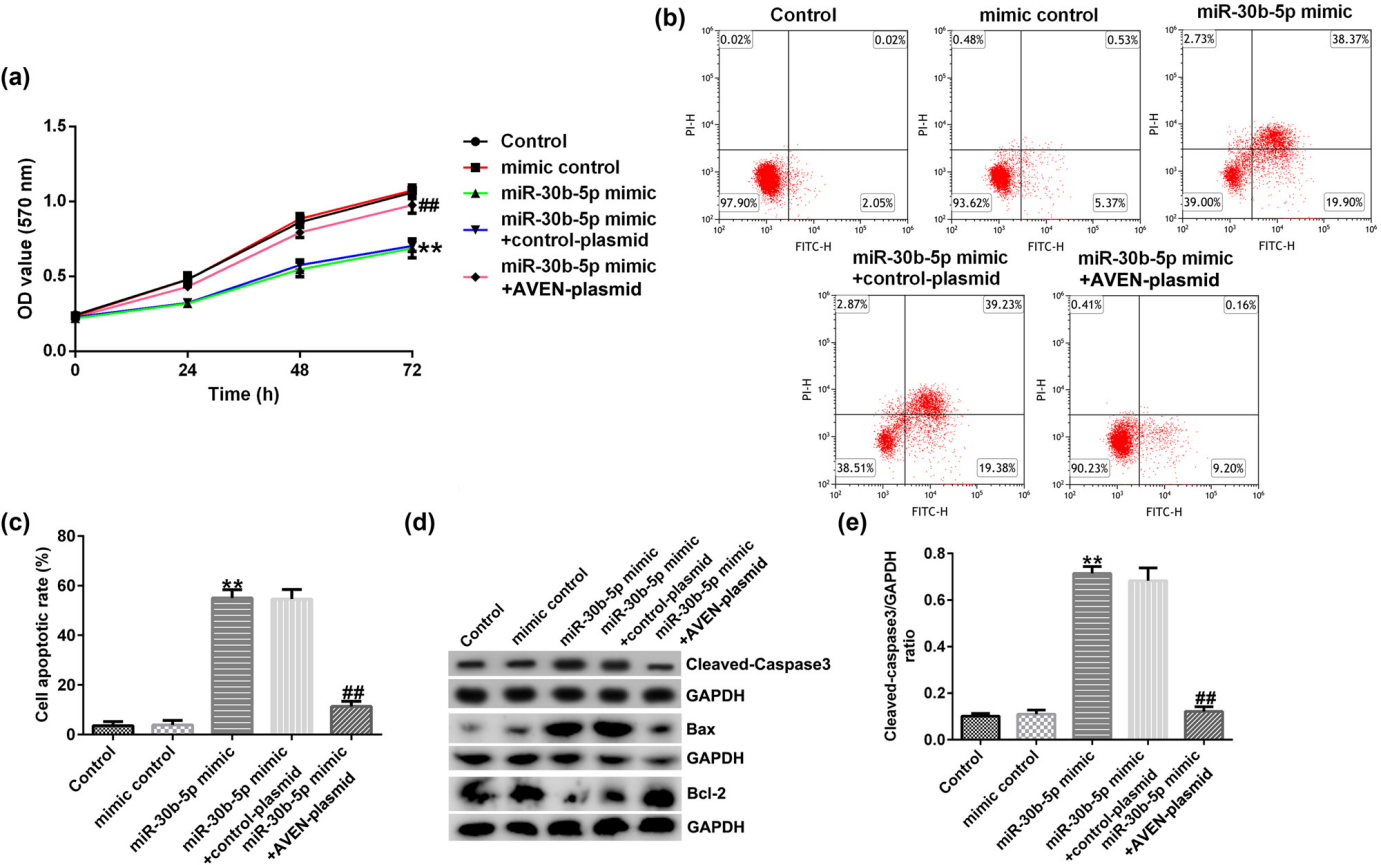
miR-30b-5p overexpression inhibits proliferation and promotes apoptosis in SW480/R via AVEN. (a) MTT assay was carried out to assess SW480/R cells viability. (b and c) FCM assay was carried to measure SW480/R cell apoptosis. (d) Western blot was used to quantify the expression levels of apoptosis-related protein (cleaved-caspase3, Bax, and Bcl-2). (e) The ratio of cleaved-caspase3/GAPDH. Data are represented as the mean value ± SD of three independent experiments. **P < 0.01 vs Mimic control; ^##^ *P* < 0.01 vs miR-30b-5p mimic + control-plasmid.

**Figure A1:**
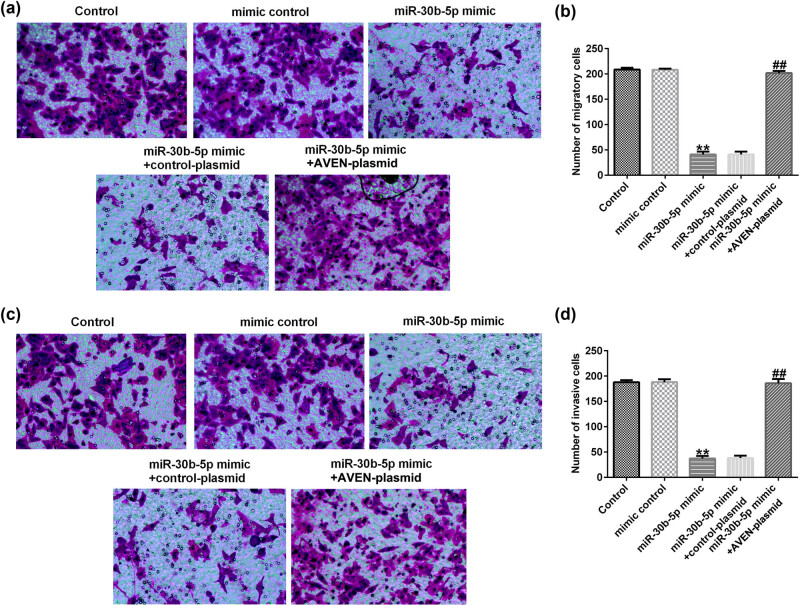
miR-30b-5p overexpression inhibits migration and invasion in SW480/R via AVEN. (a–d) Transwell assay was carried to carried to measure SW480/R cells migration and invasion. Data were represented as the mean±SD of three independent experiments. **P <0.01 vs. Mimic control; ##*P* <0.01 vs. miR-30b-5p mimic+control-plasmid.

We co-transfected miR-30b-5p mimic and AVEN-plasmid into SW480/R cells. The data depicted that cell viability was strikingly suppressed by miR-30b-5p mimic, whereas it was reversed by AVEN-plasmid (Figure 4a). Besides, cell apoptosis was specially increased by miR-30b-5p mimic, whereas it was alleviated by AVEN-plasmid (Figure 4b and c). Additionally, we detected key proteins (cleaved-caspase3, Bax, and Bcl-2) associated with apoptosis. Results indicated that the pattern of cleaved-caspase3 and Bax was dramatically upregulated, and Bcl-2 protein expression was downregulated in miR-30b-5p mimic transfected SW480/R cells, while co-transfection of AVEN overexpression plasmid decreased the level of cleaved-caspase3 and Bax, and enhanced Bcl-2 protein expression (Figure 4d and e). Moreover, we investigated the effects of miR-30b-5p and AVEN on SW480/R cell migration and invasion. Transwell assay showed that the number of migrated/invaded cells decreased when miR-30b-5p mimic was transfected in SW480/R cells, while such inhibition was reversed when co-transfected with AVEN plasmid (Figure A1a–d).

**Figure 5:**
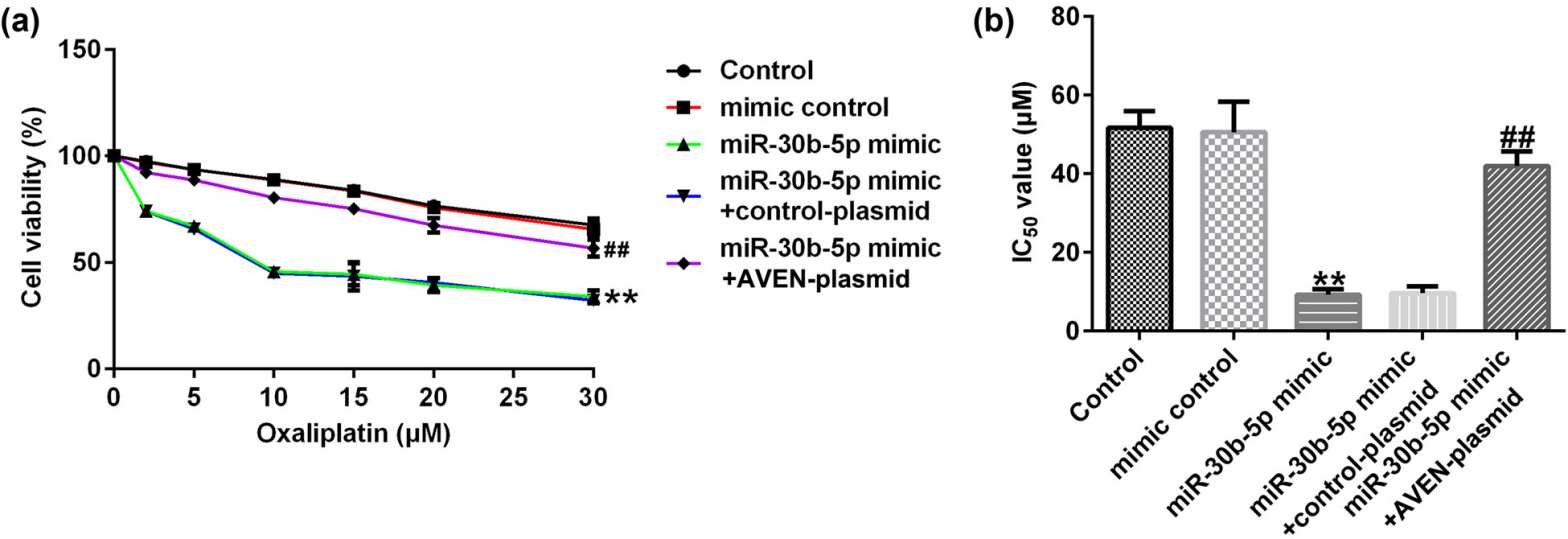
Overexpression of miR-30b-5p represses the resistance of SW480/R cells to oxaliplatin. (a) The cell viability was detected by MTT assay. (b) IC_50_ value of SW480/R cells treated with miR-30b-5p mimic. Data are represented as the mean value ± SD of three independent experiments. **P < 0.01 vs mimic control; ^##^ *P* < 0.01 vs miR-30b-5p mimic + control-plasmid.

Meanwhile, we verified the effects of miR-30b-5p on the sensitivity of SW480/R to oxaliplatin. The results of MTT assay revealed that cell viability of miR-30b-5p mimic groups were lower than mimic control groups upon different concentrations of oxaliplatin from 0 to 30 μM treatment, and the IC_50_ value was lower compared to mimic control groups, the aforementioned results were rescued by AVEN overexpression (Figure 5a and b).

### 3.5 MiR-30b-5p was upregulated in Tan IIA treated SW480/R cells

**Figure 6:**
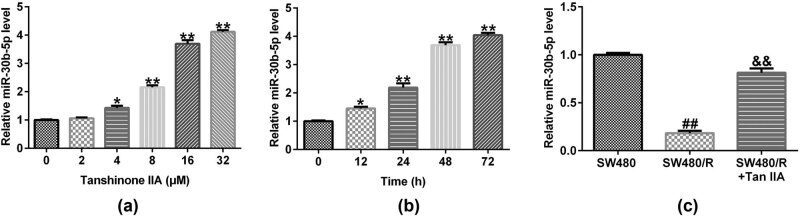
miR-30b-5p is upregulated in Tan IIA simulated SW480/R cells. (a) RT-qPCR assay was performed to detect the miR-30b-5p expression level of different dosages of Tan-IIA-treated SW480/R cells. (b) RT-qPCR assay was conducted to appraise the level of miR-30b-5p in SW480/R cells following Tan IIA (16 μM) treatment for different time points. (c) RT-qPCR assay was conducted to appraise the level of miR-30b-5p in SW480 cells, SW480/R cells, and SW480/R cells treated with 16 μM Tan IIA for 48 h. Data are represented as the mean value ± SD of three independent experiments. *, **P < 0.05, 0.01 vs Control group; ^##^ *P* < 0.01 vs SW480 cells; ^&&^ *P* < 0.01 vs SW480/R cells.

We treated SW480/R cells with different concentrations of Tan IIA from 0 to 32 μM for 48 h, and also treated SW480/R cells with 16 μM of Tan IIA at different time points from 12 to 72 h. RT-qPCR results showed that miR-30b-5p was upregulated by Tan IIA treatment in a the time-dependent and concentration-dependent manner (Figure 6a and b). Moreover, the data indicated that compared with the SW480 cells, the level of miR-30b-5p significantly reduced in SW480/R cells, while this reduction was significantly reversed by Tan IIA treatment (16 μM for 48 h) (Figure 6c). The findings suggested that Tan IIA treatment could significantly restore miR-30b-5p in SW480/R to a comparable level in SW480 cells.

### 3.6 The effect of Tan IIA in SW480/R was inhibited by miR-30b-5p inhibitor

**Figure 7:**
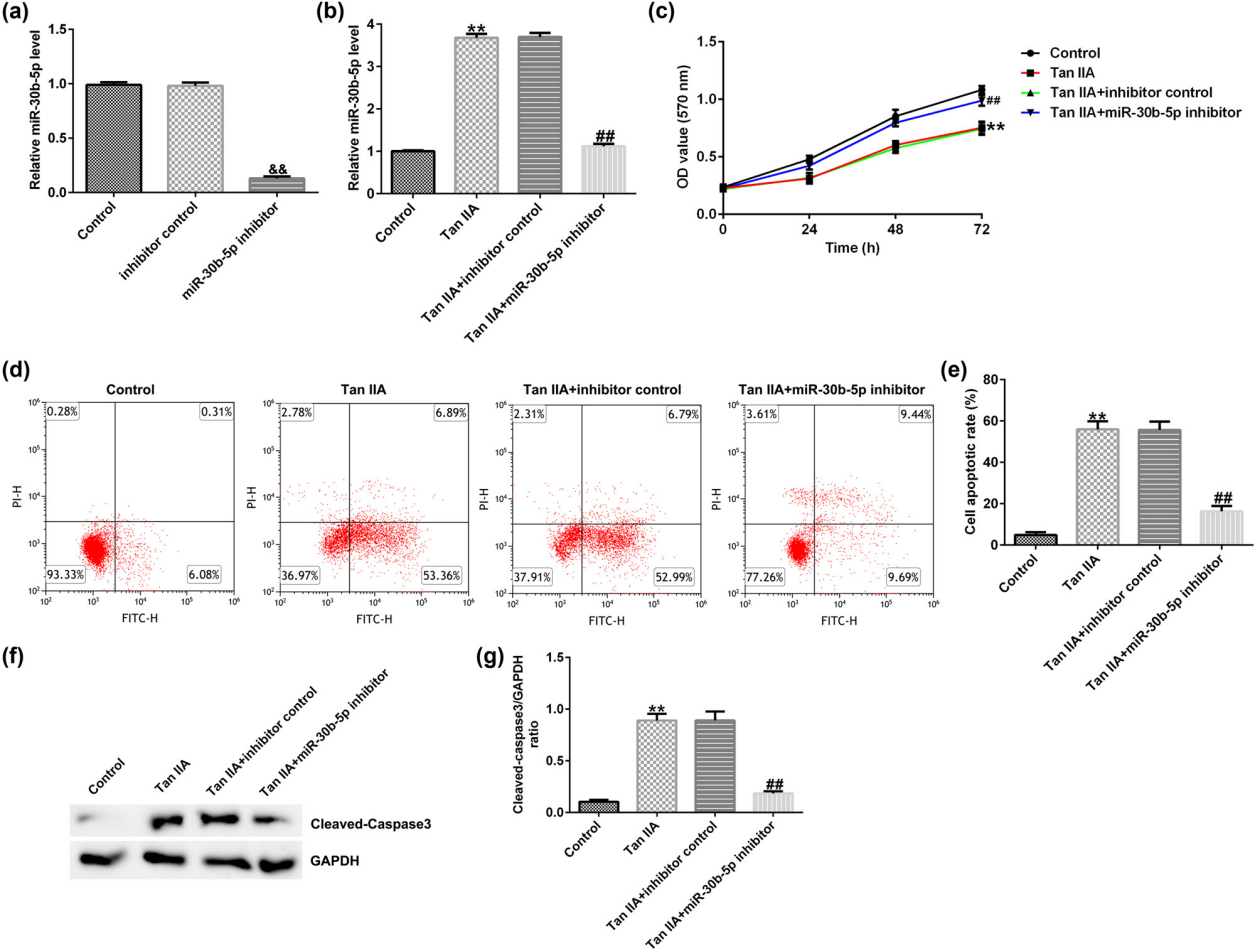
Downregulation of miR-30b-5p reverses the inhibition of SW480/R cells induced by Tan IIA. (a) The efficiency of miR-30b-5p inhibitor was verified by RT-qPCR assay. (b) RT-qPCR assay was conducted to appraise the level of miR-30b-5p in SW480/R cells following Tan IIA (16 μM) treatment for 48 h. (c) MTT assay was carried to assess SW480/R cells viability. (d and e) FCM assay was carried out to measure SW480/R cells’ apoptosis. (f) Western blot was used to quantify the expression levels of apoptosis-related protein (cleaved-caspase3). (g) The ratio of cleaved-caspase3/GAPDH. Data are represented as the mean value ± SD of three independent experiments. **P < 0.01 vs Control group; ^##^ *P* < 0.01 vs Tan IIA + inhibitor control group; ^&&^ *P* < 0.01 vs Inhibitor control group.

RT-qPCR, MTT assay, transwell assay, FCM assay, and western blot were performed to explore the relationship of Tan IIA and miR-30b-5p. First, RT-qPCR revealed that miR-30b-5p inhibitor knocks down its expression in SW480/R cells in contrast with inhibitor control groups (Figure 7a). Compared to control groups, miR-30b-5p expression level was upregulation in 16 μM Tan IIA stimulated for 48 h, but it was reduced when miR-30b-5p inhibitor was co-transfected (Figure 7b). MTT assay results showed that the inhibition effect of Tan IIA on SW480/R cells’ cell viability was rescued by miR-30b-5p inhibitor (Figure 7c). FCM assay revealed that Tan IIA caused cell apoptosis, and miR-30b-5p inhibitor transfected into Tan IIA treated SW480/R cells could decrease apoptosis (Figure 7d and e). Additionally, the protein level of cleaved-caspase3 was obviously upregulated in Tan-IIA-induced cells relative to control group, miR-30b-5p knockdown substantially reduced the level of cleaved-caspase3 (Figure 7f and g).

**Figure A2:**
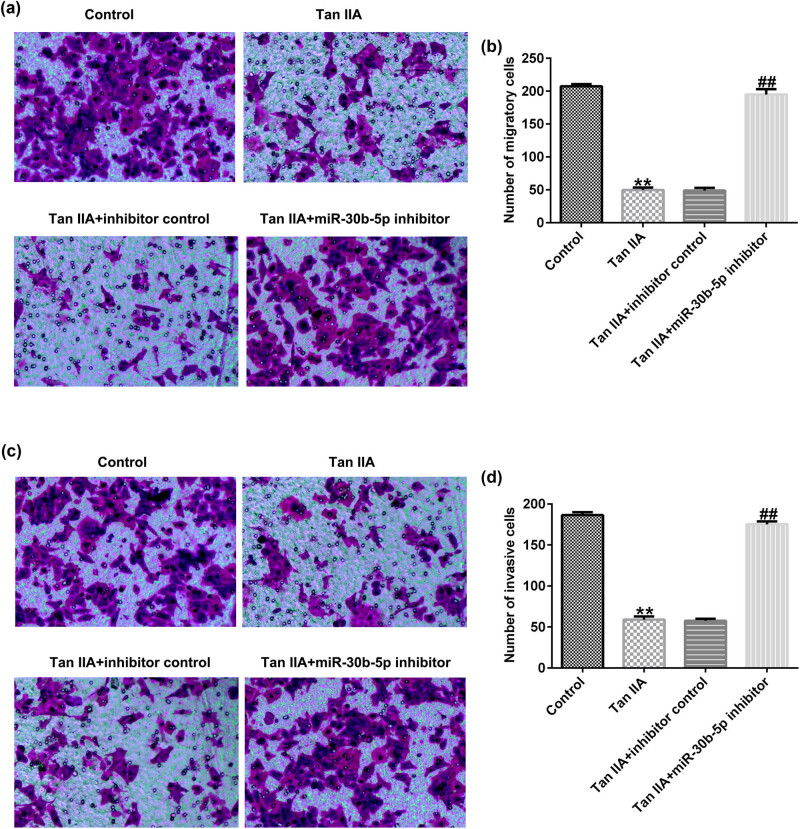
Downregulation of miR-30b-5p reveres the migration and invasion inhibition of SW480/R cells induced by Tanshinone IIA. (a–d) Transwell assay was carried to carried to measure the number of SW480/R migration and invasion cells. Data were represented as the mean±SD of three independent experiments. **P <0.01 vs. Control group; ##*P* <0.01 vs. Tan IIA+inhibitor control group.

Moreover, transwell assay showed that the number of migrated/invaded cells decreased when treated with Tan IIA, and retrieved when co-transfected with miR-30b-5p inhibitor (Figure A2a–d).

### 3.7 MiR-30b-5p enhanced oxaliplatin sensitivity of Tan IIA-treated SW480/R

**Figure 8:**
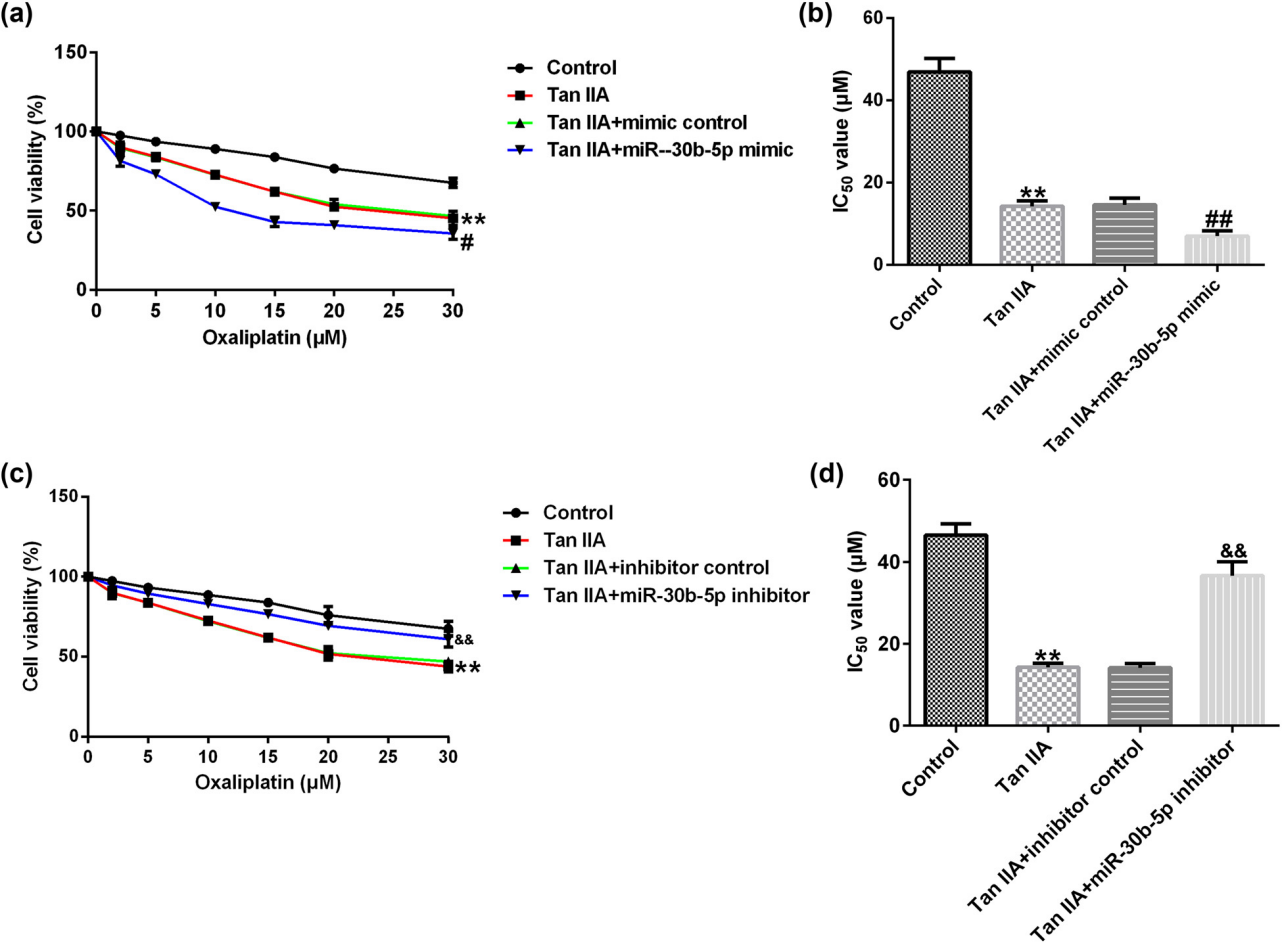
miR-30b-5p promotes the chemosensitivity of oxaliplatin induced by Tan IIA. (a) The SW480/R cell viability was detected by MTT assay with different concentrations of oxaliplatin stimulated after Tan IIA treatment and miR-30b-3p mimic transfection. (b) IC_50_ value of SW480/R cells. (c) MTT assay was carried out to assess cell viability of SW480/R with different concentrations of oxaliplatin stimulated after Tan IIA treatment and miR-30b-3p inhibitor transfection. (d) IC_50_ of SW480/R cells. Data are represented as the mean value ± SD of three independent experiments. **P < 0.01 vs control group; ^#, ##^ *P* < 0.05, 0.01 vs Tan IIA + mimic control group; ^&&^ *P* < 0.01 vs Tan IIA + inhibitor control group.

To research the effect of miR-30b-5p in oxaliplatin sensitive to Tan IIA treated SW480/R cells, SW480/R cells were treated with 16 μM Tan IIA and co-transfected with miR-30b-5p mimic or miR-30-5p inhibitor, then were treated with different dosages of oxaliplatin for 48 h. MTT analysis certified that cell viability of Tan-IIA-induced SW480/R cells was lower than that in the control group, and IC_50_ value also decreased, the phenomenon was strengthened by miR-30b-5p mimic co-transfection (Figure 8a and b).

Conversely, MTT analysis revealed that cell viability of Tan-IIA-induced SW480/R cells was lower than that of control group, and IC_50_ value also decreased, the phenomenon was reversed by miR-30b-5p inhibitor co-transfection (Figure 8c and d).

## 4 Discussion

Currently, CRC is the common malignant tumors in the world [31]. Oxaliplatin is the first-line chemotherapy drug for CRC patients [32]. Therefore, to study the molecular mechanism underlying oxaliplatin resistance in CRC and verify novel therapeutic targets for prevention of oxaliplatin resistance are imminent. In this research, we elucidated that the relationship of Tan IIA and miR-30b-5p enhanced the sensitivity of oxaliplatin in SW480/R cells.

Recent data have revealed that Tan IIA had anti-chemoresistance activity of various cancer cells. Li and Lai reported that Tan IIA could enhance the sensitivity to doxorubicin in doxorubicin-resistant breast cancer cells [33]. Guo et al. indicated that Tan IIA could enhance the effect of imatinib in leukemia mouse [34]. Meanwhile, Tan IIA increased the sensitivity of lung cancers to cyclophosphamide [35]. However, few research works were done to dissect the role of Tan IIA in oxaliplatin-resistant CRC. In our research, we found that treatment of Tan IIA significantly inhibited the proliferation of oxaliplatin-resistant CRC cells and enhanced the sensitivity to oxaliplatin.

MiRNAs are a short, non-coding RNA which affect expression of target by inhibiting translation or degrading messenger RNA [36]. There is growing evidences that miRNAs play a critical role in the drug resistance of cancer. Liu et al. found that miR-128-3p increases chemosensitivity of oxaliplatin-resistant CRC [37]. Zhao et al. demonstrated that miR-200c played a critical role in paclitaxel-resistance in human lung cancer A549 cells [38]. In this study, we expounded that the expression level of miR-30b-5p was clearly decreased in SW480/R cells. And consistent with previous study [29], we confirmed that AVEN is a direct target of miR-35b-5p. miR-35b-5p overexpression could inhibit the proliferation, migration, and invasion of SW480/R cells, and enhanced cell apoptosis by targeting AVEN. Importantly, miR-35b-5p overexpression enhanced oxaliplatin sensitivity in SW480/R cells. A recent study indicated that miR-30c is associated with inflammation by Tan IIA on atherosclerosis [39]. In this study, we found that Tan IIA treatment significantly enhanced miR-30b-5p expression in SW480/R cells, and miR-30b-5p inhibition significantly reversed the inhibitory effects of Tan IIA on the malignant biological behaviors of SW480/R cells. However, the possible mechanisms of how Tan IIA treatment leads to upregulation of miR-30b-5p remains to be investigated. And whether other miR-30 family members (such as miR-30c) [40] could be regulated by Tan IIA treatment in CRC cells is unclear. These were the limitations of current study and we will investigate these issues in the future. Finally, the findings of present study revealed that miR-30b-5p promotes the inhibitory effect of Tan IIA on oxaliplatin resistance in SW480/R cells.

## 5 Conclusion

miR-30b-5p markedly inhibited the malignant biological behaviors of SW480/R cells by targeting AVEN, and Tan IIA inhibited the malignant biological behaviors of SW480/R cells via the upregulation of miR-30b-5p. Tan IIA significantly enhanced the sensitivity of SW480/R cells to oxaliplatin, and this enhancement could be further promoted by miR-30b-5p upregulation. Therefore, miR-30b-5p combined with Tan IIA may be a new therapeutic strategy for the treatment of oxaliplatin-resistant CRC.
